# Loss formulations for assumption-free neural inference of SDE coefficient functions

**DOI:** 10.1038/s41540-025-00500-6

**Published:** 2025-03-01

**Authors:** Marc Vaisband, Valentin von Bornhaupt, Nina Schmid, Izdar Abulizi, Jan Hasenauer

**Affiliations:** 1 Department of Internal Medicine III with Haematology, Medical Oncology, Haemostaseology, Infectiology and Rheumatology, Oncologic Center, Salzburg Cancer Research Institute - Laboratory for Immunological and Molecular Cancer Research (SCRI-LIMCR), Salzburg, Austria; 2 Paracelsus Medical University, Cancer Cluster Salzburg, Salzburg, Austria; 3 Bonn Center for Mathematical Life Sciences, Life & Medical Sciences (LIMES) Institute, University of Bonn, Bonn, Germany

**Keywords:** Computer modelling, Differential equations, Dynamical systems, Stochastic modelling

## Abstract

Stochastic differential equations (SDEs) are one of the most commonly studied probabilistic dynamical systems, and widely used to model complex biological processes. Building upon the previously introduced idea of performing inference of dynamical systems by parametrising their coefficient functions via neural networks, we propose a novel formulation for an optimisation objective that combines simulation-based penalties with pseudo-likelihoods. This greatly improves prediction performance compared to the state-of-the-art, and makes it possible to learn a wide variety of dynamics without any prior assumptions on analytical structure.

## Introduction

### Stochastic differential equations

*Stochastic differential equations* (SDEs) are a class of equations widely used to describe continuous-time dynamical systems, including many complex biological and natural processes. The most commonly studied form, also known as an *Itô equation*, can be represented as

1 $$d{X}_{t}=a(t,{X}_{t})dt+b(t,{X}_{t})d{B}_{t}$$

where *a* is the *drift* coefficient governing deterministic dynamics, *b* is a *diffusion* coefficient representing the variance of noise impacting the evolution of the system, and *B*_*t*_ represents Brownian Motion (see^1,2^ for a rigorous account on stochastic integration). Intuitively, they can be understood as a middle way between two contrasting levels of modelling granularity: Microscopic interacting particle systems can accurately depict individual variability but are often computationally intractable. By contrast, macroscopic ODE modelling, is computationally and theoretically well-understood, but may provide insufficient information about intra-population variance. From this view, SDEs represent a mesoscopic scaling regime where the influence of many small random interactions is summarised into the diffusion term, but not eliminated fully. This makes them an attractive choice for biological modelling^3–5^ as well as other fields such as engineering^6^, epidemiology^7^ or finance^8^.

While the study of parametric SDE inference is deeply developed, the problem of recovering coefficient functions from data without any assumptions on the analytical structure has received far less attention. Research in this direction is being done using neural networks, whose universal approximation property and ease of optimisation have in the recent past driven a highly active area of research on the intersection of deep learning and dynamical systems theory^9–11^. It broadly falls into two philosophical categories:

One view, often referred to as the *neural differential equation*^12,13^ approach, sees time series data as realisations of a generative neural network, which thus represents not just analytical dynamics but instead a sort of “solution operator”. Here, deep learning can serve as a tool towards performing inference^14^ on, or sampling from a differential equation system. Notably, Ryder et al.^15^ considered trajectories as samples from a variational RNN; more recently, Kidger et al.^16^ proposed the *SDE-GAN* method for re-interpreting them as samples from a generative adversarial model. In all these cases, the goal is to obtain a system that can produce new sample paths sampled from the SDE underlying the training data - in other words, to sample from the solution distribution. For a comprehensive treatise on these and related methods, we refer the reader to the highly influential work by Kidger^13^.

The other view, from which we undertake the proposed study, instead emphasises the role of a neural network model to fill in unknown elements of some predefined dynamical system. Pioneered by Rackauckas et al.^17^, and termed *universal differential equations*, its key aim is to enable discoveries about underlying models, for which deep learning is an instrument; in this interpretation, neural networks represent functions, not full systems^18,19^. For ordinary differential equations, and under certain assumptions on the involved kinetics, the use of deep learning has been proposed to facilitate parameter estimation^19,20^, but also for dynamics discovery^21^ via symbolic regression.

**Fig. 1:**
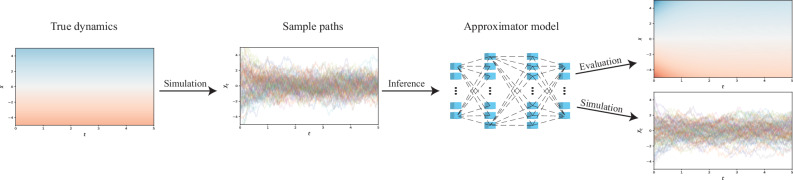
The general workflow for training a neural network to approximate the kinetics of a dynamical system. Observed data serves as the basis for inference on the underlying coefficients.

For SDEs, however, there is little established work beyond that of Rackauckas et al.^17^, who represented coefficient functions as a neural network, and subsequently optimised it by penalising disagreement between observed data and simulations from the current coefficient model candidate. It is this approach upon which we aim to improve (see also Fig. 1 for an overview), as we

- present the novel idea of utilising pseudo-likelihoods – already a well-regarded tool in parametric inference^22^ – as an alternative objective function,
- suggest more robust loss formulations for the existing, simulation-based, methods, and
- show that the two approaches can successfully be hybridised to greatly improve on the performance of either one.

As working with SDEs is a universally challenging endeavour, we lay no claim to having produced a universally optimal approach to SDE inference. However, we hope to demonstrate that the ideas we propose already provide a significant improvement on existing methods – as demonstrated by extensive benchmarking – and have great potential for the further development of robust inference algorithms.

## Results

### Combining simulation-based and pseudo-likelihood objectives yields a flexible hybrid formulation

In this study, we propose the *Hybrid Lie-Trotter* loss for a neural network representing the coefficient functions, derived in detail in section 4, whose components are

1. the Wasserstein distance between observed and re-generated samples’ empirical distributions,
2. a negative log-pseudo-likelihood of the observed sample paths given the model, and
3. the discrepancy between observed a re-generated samples’ empirical auto-correlation.

In the following, we present the result of all evaluation metrics to compare the proposed Hybrid Lie-Trotter scheme and the respective state-of-the-art, – Moment matching as proposed by Rackauckas et al.^17^ for all three metrics taking the ground truth into account, and SDE-GAN^16^ for the last, comparing solution distributions only. We examine a number of datasets, described in detail in section 4. In all tables, we abbreviate Ornstein-Uhlenbeck as OU, Cox-Ingersoll-Ross as CIR, the Unstable Sine problem as SIN1, and the Multi-Modal Sine problem as SIN2. The biological GFP transfection dataset is abbreviated GFP. In all cases, an asterisk denotes the state-of-the-art method, and the best scores are shown in boldface.

### Hybrid loss functions outperform other approaches for coefficient reconstruction

To assess the studied models’ capacity for accurately inferring drift and diffusion coefficients of paths from data, we evaluated the expected *L*^2^ error of model predictions compared to the ground truth. We found that the proposed hybrid loss yielded results that are significantly better than the state-of-the-art moment matching method (see Table 1).

**Table 1:** Mean squared reconstruction error on the drift and diffusion

	Drift				Diffusion			
Method	OU	CIR	SIN1	SIN2	OU	CIR	SIN1	SIN2
Hybrid Lie-Trotter	**0.04**	**0.12**	**0.14**	**0.11**	**0.001**	**0.002**	**0.002**	**0.003**
Moment matching*	3.65	6.58	36.29	1.18	0.191	0.495	0.143	0.456

Bold values indicate the best performance.

Between the different combinations of loss terms that we studied, we found hybrid schemes to outperform lone pseudo-likelihood-based and lone simulation-based losses in all cases. For a detailed summary of all scores for drift and diffusion coefficient reconstruction, see supplemental Tables S1 and S2 respectively.

Evaluating the results of the diffusion coefficient estimation was more difficult as most methods very accurately approximated the diffusion coefficient functions. Here, too, however, the proposed hybrid scheme – while not technically the best for any problem – always had a performance nearly indistinguishable from the best model.

### The proposed method provides higher accuracy for newly generated samples

**Fig. 2:**
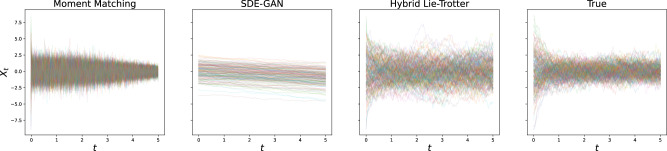
Comparison of re-sampled paths for the Ornstein-Uhlenbeck problem. Results after optimisation are shown for the state-of-the-art as well as the proposed method, and compared to the true test set.

**Fig. 3:**
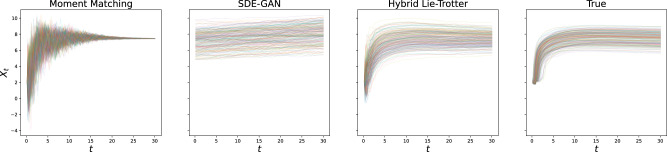
Comparison of re-sampled paths for the GFP data. Again, results after optimisation are shown for the state-of-the-art as well as the proposed method, and compared to the true test set.

For the problem of generating samples from the solution distribution, we performed the experiment of generating sample paths with either true or estimated dynamics, and the same initial condition and driving Brownian Motion sample paths. Here, too, we reported the *L*^2^ error and found the hybrid schemes to fare much better than individual losses, and in particular than moment matching (see Table 2; as well as, for example, Figs. 2 and 3 for the visualisations of the results on the Ornstein-Uhlenbeck and GFP data, respectively).

**Table 2:** Mean squared reconstruction error on newly generated paths vs. true dynamics, driven by the same Brownian Motion

Method	OU	CIR	SIN1	SIN2
Hybrid Lie-Trotter	**0.56**	**2.70**	**3.21**	**24.36**
Moment matching*	1.94	4.61	15.86	55.61

Bold values indicate the best performance.

As before, in the comparison of all loss combinations, no one method proved best for all problems (see supplemental Table S3 for a full breakdown of achieved scores), but hybrid approaches always outperformed moment matching. In our final evaluation, we evaluated the signature transform metric to also allow comparisons to the SDE-GAN method and for problems without a ground truth. With respect to this metric, too, we found that the proposed scheme outperformed the state-of-the-art (for the detailled results, see supplemental Table S4).

From the perspective of purely sampling from the solution distribution, measured in quality by maximum mean discrepancy from testing samples after depth-5 signature feature transform, there was a very wide spread of scores across problems and methods, but the proposed loss functions combining pseudo-likelihoods, the Wasserstein, and the auto-correlation losses outperformed the state-of-the-art SDE-GAN method in all cases (see Table 3).

**Table 3:** Depth-5 signature transformed Maximum Mean Discrepancy metric, as proposed in the evaluation of^16^

Method	OU	CIR	SIN1	SIN2	GFP
Hybrid LT	**5.00**	**9.73**	**49.98**	**444.39**	**1.54**
SDE-GAN*	13.58	20.59	80.35	5.80e+4	64.79

Bold values indicate the best performance.

### Simulation time is a decisive contribution to computational cost

Evaluating the runtimes of the different methods, we could – unsurprisingly – observe that methods that generate synthetic samples as part of the loss function evaluation take the largest computational effort to train (for a full runtime comparison, see Table 4). Compared to the Euler-Maruyama pseudo-likelihood, the calculation of second derivatives that is necessary for the Lie-Trotter scheme creates additional overhead, but its magnitude is insignificant compared to the simulation overhead (of course this depends strongly on the number of trajectories generated in each optimiser step, but should be true for any reasonable number of synthetic paths).

**Table 4:** ${\log }_{10}$ fold change of runtimes as compared to the state-of-the-art “Moment” loss

Method	Runtime log10FC vs. Moment matching*
Euler-Maruyama	−2.60
Lie-Trotter	−1.54
Wasserstein	0.16
Correlation	0.02
Euler-Maruyama + Moment	0.04
Euler-Maruyama + Wasserstein	0.20
Euler-Maruyama + Correlation	0.07
Lie-Trotter + Moment	0.14
Lie-Trotter + Wasserstein	0.29
Lie-Trotter + Correlation	0.16
Euler-Maruyama + Moment + Correlation	0.05
Euler-Maruyama + Wasserstein + Correlation	0.20
Lie-Trotter + Moment + Correlation	0.16
Lie-Trotter + Wasserstein + Correlation	0.30

A direct comparison of runtime to the SDE-GAN approach was not possible, as the methods were executed on different machines. However, we can confidently say that the SDE-GAN training is more computationally intensive by orders of magnitude, as 100 epochs of training required approximately 68 hours on a GPU node in a computing cluster, while all other models trained in less than two hours on a consumer-grade CPU.

## Discussion

In this study, we investigated the problem of inferring SDE coefficient functions from data given no assumptions on analytical structure. We introduced the existing methods and the core idea of representing the structurally unknown dynamics of a differential equation system by a deep neural network, and proposed a novel objective function formulation, which combines strategies from simulation-based inference and classical approximation methods, penalising both discrepancies between observed and re-simulated data, and the negative pseudo-likelihood induced by the Lie-Trotter scheme. The advantages of its combined formulation can be gleamed by considering the issues faced by the two respective loss function concepts: The simulation losses that penalise discrepancies of marginal distributions efficiently control population summary statistics, but the behaviour of individual paths can become extremely erratic; pseudo-likelihood optimisation, by contrast, controls the behaviour of individual sample increments, but can infer no information in areas of phase space with little or no data, leading to the danger of diverging sample paths. Thus, the two approaches, when hybridised, efficiently address each other’s shortcomings (see supplemental Fig. S1 for an illustration of the regularisation provided by increasing the weight of the Wasserstein term). As a result, the evaluation on both synthetic and real-life data showed that this combined loss formulation consistently performs better than the state of the art, and qualitatively captured the dynamics of the different datasets well.

The next step towards using the presented system for obtaining biological insights would be to identify analytical structure behind the data, leading to the field of symbolic regression. Initial experimentation with a method recently proposed by Cranmer et al.^23^ has proven promising on the simple Ornstein-Uhlenbeck example; but has so far not resulted in reliable mechanics identification for the more complex problems, suggesting a need for further investigation. To make the results of dynamics discovery more interpretable and reliable, uncertainty quantification on the estimated coefficient functions would be of great value, e.g. using Bayesian Neural Networks^24^. We have not been able to use them to produce consistently robust fits, but believe that technological advances may overcome this limitation. For the SDE-GAN method, we observed a broad failure to train with the procedure and hyperparameters in^16^, despite copying the original setup, observing an imbalance in training as the generator loss continuously increased – we hypothesise that this could be due to vanishing gradients if the generator initialisation is too far away from the true distribution and it cannot produce credible sample paths; however we were not able to pinpoint the exact reason, or obtain a better hyper-parameter configuration.

All experiments we considered, including the real-life biological data, were in a dense-data regime. More research is needed on possible adaptations to data sparsity, which occurs often in applications. Given larger time steps, we would expect them to impact the less stable Euler-Maruyama pseudo-likelihood more strongly than the other loss components. To build a system more robust towards data sparsity, however, there are a number of possibilities. The most promising, in our opinion, would be to co-opt strategies often utilised in classical parametric inference, such as bridge re-sampling approaches^22,25^ which repeatedly iterate between re-sampling paths based on the current model and updating the model based on the imputed data. While in our study we utilised a simple forward scheme for simulating sample paths within the loss function evaluation – in order for it to remain compatible with automatic differentiation and thus the neural network training –, future work would certainly benefit from the use of automatically differentiable implementations of higher-order solvers, such as those in the growing Diffrax library^13^. Given sufficient data density, on the other hand, another promising avenue would be de-coupling the tasks of estimating the drift and diffusion, thus utilising a timescale separation approach, as has been suggested by Ditlevsen in the context of climate modelling^26^.

The overall evaluation of our results showed, most of all, that SDE inference without any structural assumptions remains a formidable challenge. At the same time, our contributions provide a significant increase in estimation quality compared to the state-of-the-art, and we see them as a stepping stone towards a more comprehensive framework for stochastic dynamics discovery from data.

## Methods

Assume that we observe a set of trajectories $X={({X}^{(i)})}_{1\le i\le N}$, of which each is sampled at a set of discrete time points ${({t}_{k})}_{k}$. They are assumed to be realisations of a strong solution to some SDE in the form of equation (1), with no prior knowledge about the analytical structure of the functions *a*, *b*. The objective is now to train a neural network to represent these unknown functions, which map from (*t*, *x*)-phase space to scalar drift or diffusion, respectively. Given a good fit, this can be used to investigate the dynamics, but also to sample anew from the solution distribution. This naturally depends on an advantageous loss function to quantify disagreement in terms of empirical moments between network predictions and actually observed data.

The most natural choice, would be to directly apply maximum-likelihood estimation: If we observe a transition from state *x* at time *t* to ${x}^{{\prime} }$ at time ${t}^{{\prime} }$, we would like to access the solution’s *transition kernelπ*, yielding our desired objective, given candidate functions $\hat{a}$, $\hat{b}$,

$$-\log {\pi }_{\hat{a},\hat{b}}(t,x,{t}^{{\prime} },{x}^{{\prime} }):=-\log {\mathbb{P}}({Y}_{{t}^{{\prime} }}\in d{x}^{{\prime} }\,| \,{Y}_{t}\in dx)$$

where *Y* solves

$$d{Y}_{t}=\hat{a}(t,{Y}_{t})dt+\hat{b}(t,{Y}_{t})d{B}_{t}$$

Unfortunately, except for very few cases, this likelihood is intractable. Even when the dynamics are structurally known, few SDEs admit an analytical solution, and fewer still an explicit transition density. In our “assumption-free” case, there is no way at all to grasp at this object – numerically or otherwise.

The avenues that remain open are similar to those in other domains and contexts where direct likelihood evaluation is impossible:

- Either circumventing it completely by using a simulator for the studied dynamical system (Methods of this kind are frequently referred to as “likelihood-free” and appear in numerous applications^27^, especially Bayesian learning^28^), or
- making do with some approximation of the likelihood, if a reasonable choice exists.

### Simulation-based learning

Employing a simulator, which in our case is any SDE solver, to address the inaccessible likelihood can be done in a straightforward fashion. By generating a number of SDE trajectories from the current network state, and comparing summary statistics with those obtained from the observed data, we can quantify the dissimilarity between the real and estimated dynamics.

To put this into formulae, let $X\in {{\mathbb{R}}}^{N\times T}$ denote the data consisting of *N* independent trajectories at *T* timepoints. Moreover, let Ξ, here and in the following, denote the SDE solution operator with some abuse of notation: Given coefficient functions *a*, *b*, we write Ξ_*m*_(*a*, *b*) to denote *m* independent paths sampled from the solution of the SDE in equation (1).

#### Moment matching

To compare simulated to real samples, Rackauckas et al.^17^ have proposed using moment matching. They suggest constructing a loss by considering timepoint-wise population means and variances

$$\begin{array}{ll}{{\mathcal{L}}}_{m}^{{\rm{Moment}}}(X;a,b):=| | \hat{{\mathbb{E}}}[X]-\hat{{\mathbb{E}}}[{\Xi }_{m}(a,b)]| {| }^{2}\\\qquad\qquad\qquad\quad\quad +| | \hat{{\mathbb{V}}}[X]-\hat{{\mathbb{V}}}[{\Xi }_{m}(a,b)]| {| }^{2}\end{array}$$

where $\hat{{\mathbb{E}}}$ and $\hat{{\mathbb{V}}}$ denote the *timepoint-wise* empirical mean and variance, respectively. Among the approaches directly recovering the coefficient functions, this is the state-of-the-art.

#### Wasserstein penalty

While the mean and variance provide a good first grasp of the properties of a probability distribution, it is well-known that distributions can have a “moment distance” of zero yet vary wildly even if they coincide in infinitely many moments^29^. This motivates us to propose the *Wasserstein metric*, which is widely considered as more robust in other applications^30^. Given some measures *μ*, *ν*, it is defined via the minimisation problem

$${{\mathcal{W}}}_{p}(\mu ,\nu ):={\left(\mathop{\inf }\limits_{\pi \in \Pi (\mu ,\nu )}\int| | x-y| {| }^{p}d\pi (x,y)\right)}^{\frac{1}{p}}$$

where *Π*(*μ*, *ν*) is the set of *couplings*, i.e. joint measures whose marginals are *μ* and *ν*, respectively. In particular for the case *p* = 1, ${{\mathcal{W}}}_{1}(\mu ,\nu )$ can be intuitively interpreted as the amount of “work” necessary to reach *ν* from *μ* (or vice versa) by shifting probability mass, and thus provides a much more holistic quantification of the distance between measures.

Similar to the moment loss formulation above, we can use it to penalise differences in finite-dimensional distributions between observed and generated samples. We opt to penalise both the average and maximum loss, so that irregularities at specific timepoints are not overshadowed by overall agreement. Thus, we set

$$\begin{array}{ll}\quad\;\,{{\mathcal{L}}}_{m}^{{\mathcal{W}}}(X;m,a,b)\\ :=\displaystyle\frac{1}{T}\sum {\hat{{\mathcal{W}}}}_{1}(X,{\Xi }_{m}(a,b))+\max ({\hat{{\mathcal{W}}}}_{1}(X,{\Xi }_{m}(a,b)))\end{array}$$

where ${\hat{{\mathcal{W}}}}_{1}$ is the *timepoint-wise* Wasserstein metric.

#### Auto-correlation matching

The previous summary statistics considered differences between the finite-dimensional distributions of observed and simulated samples, i.e. a population’s collective properties at discrete time points. An alternative approach is to consider trajectories’ empirical relation. Intuitively, while marginal sample variance will describe the typical variation experienced by a population, the auto-correlation will instead measure the typical variation experienced by an *individual*. This promises to alleviate an often-seen problem with underestimating the diffusion coefficient’s magnitude when the drift is “too flexible” and can itself explain a majority of the (in truth random) variation.

We define, for some lag *k* and truncation parameter *K*, the *lag-k*-*autocorrelation*

$$\begin{array}{ll}{\rho }_{k}({X}^{(i)})\,:=\,{\rm{Corr}}\left({({X}_{j}^{(i)},{X}_{j+k}^{(i)})}_{1\le j < T-k}\right)\\\quad {\rho }_{k}(X)\,:=\,\displaystyle\frac{1}{N}\sum\limits_{1\le i\le N}{\rho }_{k}({X}^{(i)})\end{array}$$

to set

$${{\mathcal{L}}}_{m}^{{\rm{AC}}}(X;m,a,b):=\sum _{1\le k\le K}{({\rho }_{k}(X)-{\rho }_{k}({\Xi }_{m}(a,b)))}^{2}$$

### Pseudo-likelihoods

While solutions to SDEs are infinite-dimensional stochastic processes, in a computational setting one uses solution algorithms to approximate them at discrete timepoints. In this context, it is reasonable to hope that the transition kernel of a solution may also be approximated by the transition kernel of an approximation scheme (if that one is in fact tractable). This idea gives rise to the concept of *pseudo-likelihoods* (which we will denote by *Λ* to avoid confusion with losses).

These expressions approximate the likelihood of observing a particular transition assuming coefficient functions *a*, *b*

$$\Lambda (t,{X}_{t},\Delta t,{X}_{t+\Delta t};a,b)\approx {\pi }_{a,b}(t,{X}_{t},t+\Delta t,{X}_{t+\Delta t})$$

and are then converted to losses by aggregating them over all transitions observed in the data. For any method *M* presented below, we will set

$$\begin{array}{ll}\quad\;{{\mathcal{L}}}^{M}(X;a,b)\\ :=\frac{1}{N(T-1)}\sum\limits_{\begin{array}{c}{\rm{obs.}}\,{\rm{transitions}}\\ {X}_{t}^{(i)}\to {X}_{{t}^{{\prime} }}^{(i)}\end{array}}-2\log {\Lambda }^{M}(t,{X}_{t}^{(i)},{t}^{{\prime} }-t,{X}_{{t}^{{\prime} }}^{(i)};a,b)\end{array}$$

#### Euler-Maruyama

The *Euler-Maruyama* scheme^31^ is the oldest and most well-known solution scheme for SDEs. It is a direct extension of the forward Euler scheme for ordinary differential equations, and relies on the approximation

$${X}_{t+\Delta t}\approx {X}_{t}+a(t,{X}_{t})\cdot \Delta t+b(t,{X}_{t})\cdot ({B}_{t+\Delta t}-{B}_{t})$$

It immediately follows that

$${X}_{t+\Delta t}-{X}_{t}\mathop{ \sim }\limits^{{\rm{approx}}}{\mathcal{N}}(a(t,{X}_{t})\cdot \Delta t,{b}^{2}(t,{X}_{t})\cdot \Delta t)$$

If we now denote by *Λ*^EM^ the explicit pseudo-likelihood of the scheme above, it holds that

$$\begin{array}{ll}\quad-2\log {\Lambda }^{{\rm{EM}}}(t,{X}_{t},\Delta t,{X}_{t+\Delta t};a,b)\\ =\log (2\pi {b}^{2}(t,{X}_{t})\Delta t)+\frac{{({X}_{t+\Delta t}-{X}_{t}-a(t,{X}_{t})\Delta t)}^{2}}{{b}^{2}(t,{X}_{t})\Delta t}\end{array}$$

#### Lie-Trotter splitting

The *Lie-Trotter splitting* scheme is an approximation algorithm for SDEs that relies on the well-known Lie product formula and was first proposed by Bensoussan et al.^32^. Its idea is to introduce auxiliary functions to split up the process evolution into a purely deterministic ODE update and the stochastic rest.

Pilipovic et al.^33^ show that it, too, possesses an explicit pseudo-likelihood and provides formulae. In our case, where the drift and diffusion are fundamentally nonlinear, they are reduced to

$$\begin{array}{lll}\quad-2\log {\Lambda }^{{\rm{LT}}}(t,{X}_{t},\Delta t,{X}_{t+\Delta t};a,b)\\ =\,\log ({b}^{2}(t,{X}_{t})\Delta t)\\ +\,\frac{{({X}_{t+\Delta t}-{X}_{t}-a(t,{X}_{t})\Delta t-\frac{{(\Delta t)}^{2}}{2}a(t,{X}_{t}){\partial }_{x}a(t,{X}_{t}))}^{2}}{{b}^{2}(t,{X}_{t})\Delta t}\end{array}$$

It thus becomes evident that for our case, this more sophisticated scheme simply introduces a second-order term into the drift approximation. For a more detailled derivation, we refer the reader to ref. ^33^.

### Combination approaches

From a conceptual point of view, the two presented classes of approaches are complementary in the sense that they address each other’s most obvious weaknesses: Pseudo-likelihood-based methods do not provide any information about the regions of phase space with no data, while simulation-based methods may not sufficiently control the local behaviour of individual trajectories as long as the overall summary statistics remain matching.

**Fig. 4:**
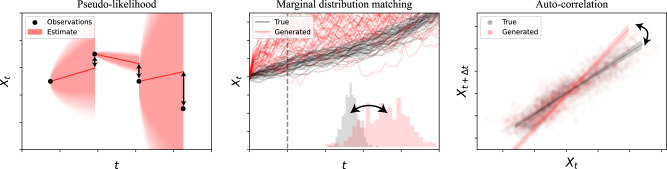
Visualisations of the different loss strategies. Pseudo-likelihood penalties aim to control the behaviour of individual increments, while simulation-based losses match population statistics.

It is therefore only natural to introduce loss functions that simply add up both simulation- and pseudo-likelihood-based penalties. Specifically, we propose a combination loss to which we will in the following refer to as *Hybrid Lie-Trotter*:

$$\begin{array}{ll}\quad\;\,{{\mathcal{L}}}_{m,\eta }^{{\rm{HLT}}}(X;m,a,b)\\ :={{\mathcal{L}}}_{m}^{{\mathcal{W}}}(X;m,a,b)+\eta {{\mathcal{L}}}^{{\rm{LT}}}(X;a,b)+{{\mathcal{L}}}_{m}^{{\rm{AC}}}(X;m,a,b)\end{array}$$

for a weighting constant *η*. Despite its simplicity, we will demonstrate that *ceteris paribus*, it consistently outperforms both the current state-of-the-art of only matching moments and alternative formulations. (For a brief visual summary of the notions behind the individual loss terms, see Fig. 4).

### Experimental evaluation

To rigorously evaluate the different methods’ performances, we study five datasets (directly randomly split into 80% training and 20% test data), of which four are synthetic and showcase different kinds of dynamics SDEs can exhibit. The fifth consists of real experimental data collected and analysed by Fröhlich et al.^34^ in the study of mRNA translation dynamics, which presents an application example of innate stochasticity in biological systems.

#### Ornstein-Uhlenbeck process

**Fig. 5:**
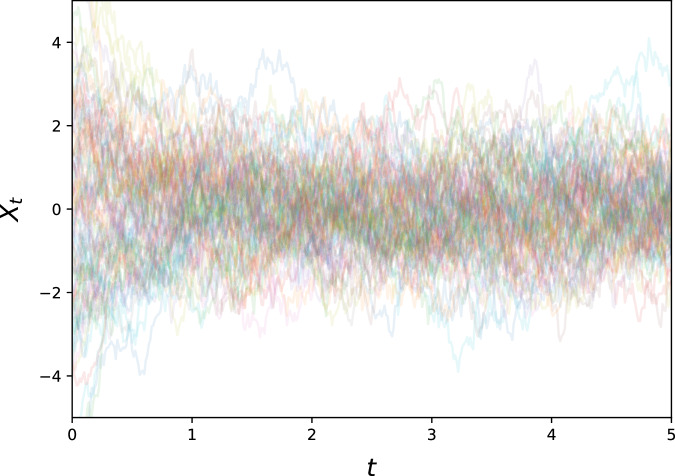
300 generated Ornstein-Uhlenbeck sample paths. This represents an archetypical noise process with a stable attractor at 0.

The Ornstein-Uhlenbeck process^35^ is one of the most emblematic examples for an SDE and can be written as

$$d{X}_{t}=-\lambda {X}_{t}dt+\sigma d{B}_{t}$$

with *λ* > 0, *σ* > 0. Note that the drift term induces exponential decay, so that the resulting process will not diverge, and is generally speaking relatively benign, having a single point of attraction at *X*_*t*_ = 0, towards which the drift will push it exponentially (see Fig. 5).

We used *λ* = 2, *σ* = 2 as example parameters, and generated 5000 paths with initial values samples from ${\mathcal{N}}(0,3)$. These paths were sampled at 400 equidistant time points for *t* ∈ [0, 5].

#### Cox-Ingersoll-Ross process

**Fig. 6:**
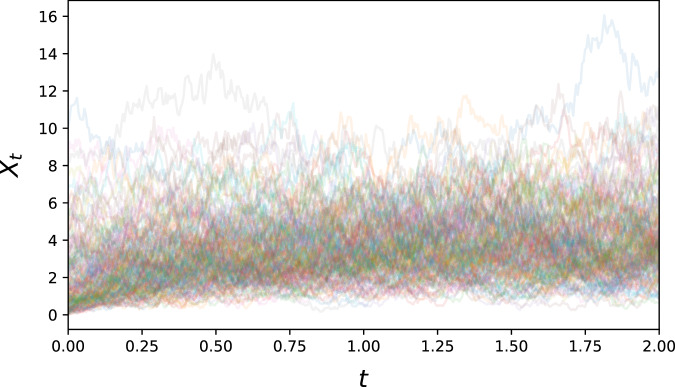
300 generated Cox-Ingersoll-Ross sample paths. This process is a classical model of volatility, with asymmetric fluctuation around its stable point.

A step up in complexity is the *Cox-Ingersoll-Ross* process first introduced by Cox, Ingersoll and Ross^36^ for the study of interest rates. It is given by

$$d{X}_{t}=-\alpha ({X}_{t}-\mu )dt+\sigma \sqrt{{X}_{t}}d{B}_{t}$$

for some *α*, *μ*, *σ* > 0, and thus notably has a non-constant diffusion term. Its drift has a stationary point at *X*_*t*_ = *μ* towards which it will exponentially gravitate, and is by construction non-negative (see Fig. 6).

For our simulation of the Cox-Ingersoll-Ross equation, we set *α* = 2, *μ* = 4, and *σ* = 2. We generated 5000 paths, with initial distribution ${\rm{Exp}}(1/2)$, sampled at 400 equidistant time points for *t* ∈ [0, 2].

#### Unstable sine

**Fig. 7:**
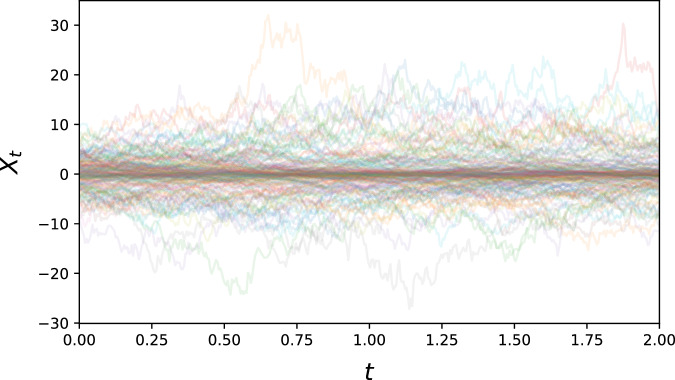
300 generated sample paths for the unstable sine problem. This dataset features increased volatility further away from the (unstable) equilibrium.

To provide a higher variety of difficult problem settings, we also study several synthetic datasets with a more challenging coefficient structure. The first is given by

$$d{X}_{t}=(\alpha \sin (\beta {X}_{t})-\gamma {X}_{t})dt+\sigma (1+| {X}_{t}| )d{B}_{t}$$

with *α*, *β*, *γ*, *σ* > 0. It combines two competing effects – *X*_*t*_ = 0 is stable with respect to the *γ*-exponential decay, but *unstable* with respect to the sine term. It also features a non-constant diffusion term (see Fig. 7).

For the simulation of the Unstable sine problem, we used the parameter values *α* = 15, *β* = (2*π*)^−1^, *γ* = 2, *σ* = 1.

#### Multi-modal sine

**Fig. 8:**
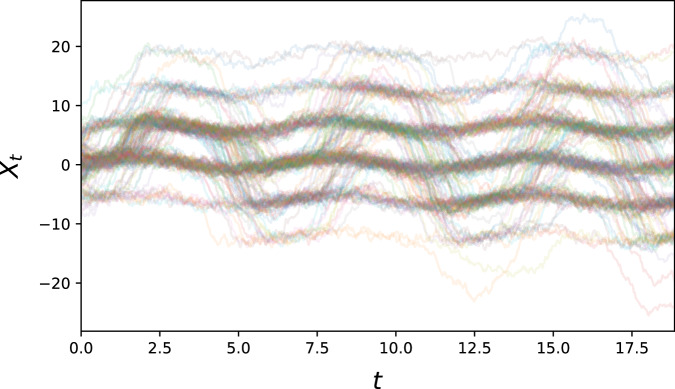
300 generated sample paths for the multi-modal sine problem. This problem gives an example of multiple time-dependent equilibria.

For a variation on the previous problem, we consider

$$d{X}_{t}=(-\alpha \sin ({X}_{t})+\beta \sin (t)-\gamma {X}_{t})dt+\sigma d{B}_{t}$$

for some *α*, *β*, *γ*, *σ* > 0. This equation now has constant diffusion, but time-dependent wells of attraction in phase space, as long as *α*/*γ* > 1. Sample paths will typically stay inside an energy well, but occasionally transition given fortuitous realisations of the driving stochastic process (see Fig. 8).

To simulate the multi-modal sine problem, we set *α* = 15, *β* = 1/(2*π*), *γ* = 2, *σ* = 1. 5000 paths with initial condition ${\mathcal{N}}(0,{3}^{2})$ were sampled at 400 equidistant time points for *t* ∈ [0, 2].

#### Biological example: GFP translation

**Fig. 9:**
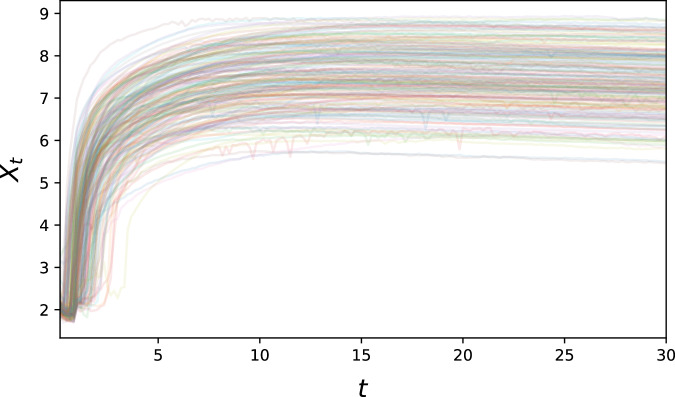
300 sample paths showing temporally resolved fluorescence of cells transfected with GFP mRNA. The observed fluorescence intensity is shown in logarithmic scale.

We also perform an evaluation using a published biological dataset^34^, which contains single-cell-resolution time-lapse data on the translation of transfected Green Fluorescent Protein (GFP) mRNA. Here, all trajectories broadly exhibit the same qualitative behaviour – an initial steep increase in fluorescence, followed by a plateau –, but a high degree of variability can be observed both on the inter-individual (e.g. in terms of maximum fluorescence) and intra-individual (exhibited by noisy behaviour over time) levels. This suggests that the translation mechanism of single cells is still on a sufficiently small scale that stochastic variation arising from its reaction kinetics can be observed directly (see Fig. 9).

### Metrics

To investigate the impact of individual components on the resulting estimates, we examined models trained with each individual loss (the simulation-based Moment and Wasserstein losses; as well as the Euler-Maruyama and Lie-Trotter pseudo-likelihoods), as well as combination losses made up from pairs of the two classes – in all cases either with or without auto-correlation matching.

To compare the quality of newly generated samples from the trained models, we also ran the SDE-GAN method, which is the state-of-the-art for generative SDE modelling, with the same set of hyperparameters as used for the non-synthetic evaluation datasets in^13^.

The question of how to assess model performance is in itself not trivial, as there are (similarly to the choice of loss function class) two philosophically different approaches: A trained network’s predictions can either be contrasted against true coefficient functions in the examples where this is possible, or evaluated based on their ability to re-generate sufficiently similar trajectories when used for SDE sampling.

#### Direct coefficient reconstruction

We aim to accurately infer unknown SDE coefficient functions from data. As such, the most natural evaluation approach is to compare the learned coefficient functions directly to the real ones. For this purpose, we sample points in phase space randomly from test data, and report the average *L*^2^ discrepancy between the true and estimated drift and diffusion values.

#### Re-sampling quality

In order to evaluate models’ capacity for re-generating samples from the correct distribution, we simulate new sample paths both from the true and estimated dynamics, using the same initial conditions and driving Brownian Motion sample paths, before comparing the *L*^2^ discrepancy.

Additionally, in order to be able to provide a comparison to the SDE-GAN method in terms of re-sampling performance, we followed the evaluation in ref. ^16^, utilising the *maximal mean discrepancy* (MMD) metric on the depth-5 *signature transform* as described by Kiraly et al.^37,38^.

### Implementation

All presented loss functions were implemented in TensorFlow^39^, using the Euler-Maruyama scheme as the simulator inside the loss evaluation. To avoid numerical issues, all data were scaled by maximal absolute value to [−1, 1] for training; the resulting model predictions were scaled back accordingly. We set the pseudo-likelihood scaling constant *η* = 10 for all problems, intended mainly to ensure that all loss terms have roughly the same magnitude. For comparing sample auto-correlation, we chose a lag cut-off at *K* = 10.

As the neural network to be trained, we chose a simple fully-connected model, taking (*x*, *t*) values as input, and consisting of four layers with **16,** **64,** **64**, and **16** neurons, respectively, and **Swish** activation. The output coefficients *a* and *b* were then represented by two output neurons using **linear** and **Softplus** activation, respectively.

Due to the computational complexity of the involved operations, we had to choose between performing comprehensive hyperparameter optimisation and testing a high number of different loss formulations. We opted for the latter, and chose to train each model with the same “common” hyperparameter setup, of:

- Optimisation using **Adam**^40^;
- an initial learning rate of 10^−3^;
- scheduled learning rate decay, by a factor of whenever an improvement in training loss of *δ* = 0.2 was not achieved after a patience period of 10 epochs (the default for TensorFlow’s ReduceLROnPlateau);
- *m* = 200 samples generated in each step for simulation losses; and
- a batch size of 2^14^ for batching the increments for the pseudo-likelihood losses.

To avoid overflows at the start of training, all models using simulator losses were pre-trained for 100 Adam steps to drift (*t*, *x*) ↦ − *x* and diffusion 1 for initialisation.

To execute the SDE-GAN method, we used the code provided by Kidger et al.^16^, which utilises PyTorch^41^. For the implementation of the signature transform and subsequent MMD metric evaluation, we used the Signatory package by Kidger et al.^42^.

## Supplementary information

Supplementary Information

## Data Availability

All data used for this study has been archived to Zenodo at https://zenodo.org/records/14784818. The biological data are from ref. ^34^ are available at https://zenodo.org/records/1228899.

## Code availability

All code used for this study has been made publicly available on GitHub, at https://github.com/marc-vaisband/LieTrotterSDE.
