# Supplementary material for: Loss formulations for assumption-free neural inference of SDE coefficient functions
Source: NPJ Syst Biol Appl. 2025 Mar 1;11:22. doi: 10.1038/s41540-025-00500-6 (PMC11873317; doi:10.1038/s41540-025-00500-6)
Supplement: Supplementary file 1 — Supplementary Information [file 41540_2025_500_MOESM1_ESM.pdf]

Supplemental Information A Loss term weight influence

In the following, a visualisation of the system's behaviour is shown upon varying the weighing constant of the Wasserstein term inside the hybrid Lie-Trotter loss. We repeated the training procedure on the Ornstein-Uhlenbeck data with different weight values for the Wasserstein term, while keeping the weights of the Lie-Trotter pseudo-likelihood, and the auto-correlation term, fixed at 10 and 1, respectively. To obtain the illustration, we then used the resulting networks for re-simulation (again with the same driving Brownian Motion sample paths, as outlined in section 4).

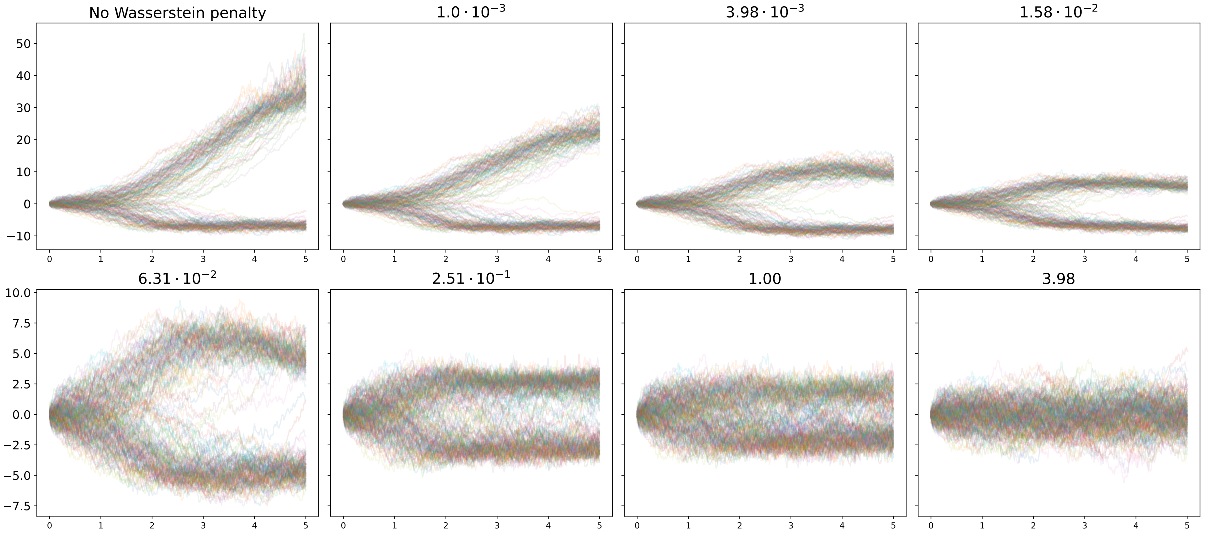

Fig. S1 Comparison of weighing constants for the Wasserstein loss term. Newly simulated samples, generated by neural network training in the Ornstein-Uhlenbeck example are shown; with different weighting coefficients of the Wasserstein penalty term in the hybrid Lie-Trotter loss. Note the different y axis scalings in the two rows.

Supplemental Information B Detailled evaluation results

Table S1 Mean squared reconstruction error on *drift* coefficients.

Method	OU	CIR	SIN1	SIN2	Rank sum
Euler-Maruyama	0.118	0.224	0.178	0.110	25
Lie-Trotter	0.117	0.225	0.216	0.110	27
Moment matching *	3.651	6.580	36.292	1.176	59
Wasserstein	2.239	3.593	25.480	1.170	55
Correlation	0.068	2.551	0.586	2.371	40
Euler-Maruyama + Moment	1.109	1.025	5.544	0.343	43
Euler-Maruyama + Wasserstein	1.693	3.304	23.707	0.858	51
Euler-Maruyama + Correlation	**0.026**	0.170	0.292	0.123	20
Lie-Trotter + Moment	0.115	0.189	0.171	**0.108**	16
Lie-Trotter + Wasserstein	0.044	0.119	0.190	0.114	16
Lie-Trotter + Correlation	0.116	0.224	0.202	0.110	25
Euler-Maruyama + Moment + Correlation	0.924	1.282	3.532	0.447	44
Euler-Maruyama + Wasserstein + Correlation	0.081	1.079	1.321	0.169	34
Lie-Trotter + Moment + Correlation	0.114	0.187	0.150	0.109	14
Lie-Trotter + Wasserstein + Correlation	0.041	**0.119**	**0.135**	0.114	**11**

Table S2 Rank sums for reconstruction error on *diffusion* coefficients in L^2 norm. Values printed as 0.000 are < 0.0005.

Method	OU	CIR	SIN1	SIN2	Rank sum
Euler-Maruyama	**0.000**	0.001	0.004	**0.002**	11
Lie-Trotter	0.000	**0.001**	0.003	0.002	9
Moment matching *	0.191	0.495	0.143	0.456	53
Wasserstein	0.233	0.342	1.530	0.321	58
Correlation	0.145	0.085	1.483	0.129	49
Euler-Maruyama + Moment	0.062	0.056	0.283	0.103	45
Euler-Maruyama + Wasserstein	0.207	0.317	1.498	0.262	54
Euler-Maruyama + Correlation	0.001	0.013	0.067	0.003	32
Lie-Trotter + Moment	0.001	0.002	0.004	0.003	19
Lie-Trotter + Wasserstein	0.001	0.002	0.005	0.003	22
Lie-Trotter + Correlation	0.000	0.001	**0.002**	0.002	**8**
Euler-Maruyama + Moment + Correlation	0.030	0.039	0.136	0.022	37
Euler-Maruyama + Wasserstein + Correlation	0.013	0.040	0.204	0.022	40
Lie-Trotter + Moment + Correlation	0.001	0.002	0.003	0.003	21
Lie-Trotter + Wasserstein + Correlation	0.001	0.002	0.002	0.003	22

Table S3 Intra-sample L^2 error based on the same realisations of Brownian Motion.

Method	OU	CIR	SIN1	SIN2	Rank sum
Euler-Maruyama	1.039e+03	1.781e+03	53.291	498.277	56
Lie-Trotter	745.857	2.722e+05	94.569	488.869	56
Moment matching *	1.938	4.608	15.858	55.614	37
Wasserstein	1.937	3.881	27.282	47.944	33
Correlation	5.052	439.685	1.058e+03	3.782e+03	53
Euler-Maruyama + Moment	0.653	0.895	14.232	33.176	20
Euler-Maruyama + Wasserstein	1.698	3.659	27.969	43.374	31
Euler-Maruyama + Correlation	0.072	32.484	9.044	36.348	25
Lie-Trotter + Moment	10.509	22.578	8.081	52.211	37
Lie-Trotter + Wasserstein	0.850	2.824	**3.151**	**23.756**	13
Lie-Trotter + Correlation	600.193	318.277	49.281	468.230	49
Euler-Maruyama + Moment + Correlation	0.636	1.032	13.935	30.336	18
Euler-Maruyama + Wasserstein + Correlation	**0.056**	**0.362**	5.247	27.005	**8**
Lie-Trotter + Moment + Correlation	10.441	16.255	6.954	52.046	33
Lie-Trotter + Wasserstein + Correlation	0.563	2.702	3.207	24.358	11

Table S4 *Sig-MMD* distance between testing data and newly generated samples. For brevity, Euler-Maruyama and Lie-Trotter are abbreviated EM and LT, respectively.

Method	OU	CIR	SIN1	SIN2	GFP	Rank sum
EM	1.584e+06	1.040e+08	8.068e+05	5.486e+06	40.999	70
LT	9.143e+05	2.793e+15	1.359e+08	1.062e+07	32.891	72
Moment	2.767	10.309	72.134	834.674	5.654	30
Wasserstein	2.784	4.564	61.697	149.641	8.762	24
Correlation	84.011	1.527e+04	4.718e+07	1.560e+08	1.969e+04	72
EM + Moment	**0.557**	4.400	69.586	589.074	5.907	19
EM + Wasserstein	3.020	**3.160**	61.689	162.529	8.906	23
EM + Correlation	1.033	4.390e+04	77.816	1.314e+04	64.280	48
LT + Moment	2.938	85.200	384.383	5.646e+03	11.943	50
LT + Wasserstein	6.122	7.130	79.000	**94.047**	14.224	37
LT + Correlation	3.902e+06	6.666e+05	2.233e+05	1.296e+07	7.525	64
EM + Moment + Correlation	1.744	4.561	204.478	246.492	4.041	24
EM + Wasserstein + Correlation	0.920	4.492	50.279	413.386	6.726	**17**
LT + Moment + Correlation	3.594	64.470	219.023	4.245e+03	9.674	48
LT + Wasserstein + Correlation	4.998	9.727	**49.981**	444.394	**1.536**	25
SDE-GAN [**?**] *	13.579	20.589	80.346	5.803e+04	64.787	57
